# Interleukin-6 as a Potential Therapeutic Target for Pulmonary Arterial Hypertension

**DOI:** 10.1155/2010/720305

**Published:** 2010-08-02

**Authors:** Yoshiaki Furuya, Toru Satoh, Masataka Kuwana

**Affiliations:** ^1^Division of Rheumatology, Department of Internal Medicine, Keio University School of Medicine, 35 Shinanomachi,Shinjuku-ku, Tokyo 160-8582, Japan; ^2^Department of Cardiology, Kyorin University Graduate School of Medicine, Mitaka, Tokyo 181-8611, Japan

## Abstract

Interleukin-6 (IL-6) is a pleiotropic cytokine with a wide range of biologic activities in immune regulation, hematopoiesis, inflammation, and oncogenesis. Recent accumulating evidence indicates a pathologic role for IL-6 in promoting proliferation of both smooth muscle and endothelial cells in the pulmonary arterioles, resulting in development of pulmonary arterial hypertension (PAH). Here, we describe a patient with mixed connective tissue disease and severe, refractory PAH. Her functional activity and hemodynamic parameters dramatically responded to tocilizumab, a humanized monoclonal antibody to human IL-6 receptor, which was aimed at treating multicentric Castleman's disease. It appears that IL-6 blockade may hold promise as an adjunct drug in treatment of PAH in idiopathic form as well as in association with connective tissue disease.

## 1. Introduction

Pulmonary arterial hypertension (PAH) is a cause of significant morbidity and mortality in patients with connective tissue disease (CTD), especially in those with systemic sclerosis (SSc) or mixed connective tissue disease (MCTD) [1]. In fact, a survival study over the past 30 years in consecutive patients evaluated at the University of Pittsburgh has demonstrated that PAH became the primary cause of SSc-related deaths today [2]. PAH is characterized by increased pulmonary vascular resistance due to remodeling of the pulmonary arterioles. Left untreated, PAH leads irremediably to right ventricular hypertrophy, pressure overload and dilation, and impaired cardiac output, resulting in death [3]. Until recently, there was no effective therapy for PAH, a disease with a median survival estimated to be approximately one year following the diagnosis in patients with SSc [4]. However, in the past two decades, novel therapies have been developed, focusing on vasoactive substances derived from the pulmonary vascular endothelium [5]. These substances, such as endothelin-1, nitric oxide, and prostacyclin regulate smooth muscle cell tone and proliferation and were shown to be central to the pathogenesis of PAH [6]. Therefore, current therapeutic agents target these 3 essential biological pathways: the endothelin-1/endothelin receptor, nitric oxide/cGMP, and prostacyclin/cAMP pathways. Improvement of symptoms, functional activity, and quality of life and even prolongation of survival have been partially achieved with currently available therapies, but mostly in patients with idiopathic PAH [5]. Indeed, it has become clearer in the past few years that SSc patients with PAH have a strikingly divergent response to current therapies and overall worse outcome compared with patients with idiopathic PAH in spite of seemingly milder hemodynamic impairment [7, 8]. In a recent multicentre longitudinal study to evaluate 3-year survival in SSc patients, 20 of 47 patients with PAH died during follow-up, giving a 3-year survival of only 56%, despite the fact that they were treated with modern PAH drugs [9]. Even in SSc patients with mildly symptomatic PAH in New York Heart Association (NYHA) functional class II, approximately two-thirds deteriorated to functional class III or IV, and some died during a 5-year period, although they were treated with one or more PAH drugs [10]. While there have been significant advances in the treatment of PAH, survival of patients with PAH associated with CTD on modern PAH drugs remains unacceptably low. Therefore, novel therapeutic strategies targeting pathways beyond pulmonary vascular endothelium are required to further improve survival of CTD patients with PAH. 

We have recently experienced a rare case of PAH-CTD complicated by multicentric Castleman's disease (MCD) during the course of the disease. MCD was successfully treated with tocilizumab, a humanized antihuman interleukin-6 (IL-6) receptor monoclonal antibody, which dramatically improved functional activity and hemodynamic parameters of PAH as well.

## 2. Case Report

A 45-year-old woman first noticed polyarthralgia and puffy fingers in 1997 and developed slowly progressive dyspnea on exertion, which made her hospitalization in a regional hospital in 2001. Pulmonary hypertension was detected by transthoracic echocardiography, which showed mild right ventricular hypertrophy in conjunction with abnormal contour of the interventricular septum and increased systolic pulmonary arterial pressure (PAP) (100 mmHg) estimated by Doppler echocardiography. Interstitial lung disease (ILD) and pericardial effusion were also detected. Taken together with increased levels of C-reactive protein (CRP), positive antinuclear, and anti-U1RNP antibodies, she was diagnosed as having mixed connective tissue disease (MCTD) complicating pulmonary hypertension. She was treated with corticosteroid pulse therapy followed by high-dose prednisolone (1 mg/kg), resulting in improvement of exertional dyspnea and reduction in estimated systolic PAP to 60 mmHg. In November 2005, she visited a pulmonologist of the referring centre because of worsening dyspnea. She underwent a systematic cardiac evaluation, including right heart catheterization and ventilation-perfusion scan, and a diagnosis of PAH in NYHA functional class III was made based on mean PAP 58 mmHg, pulmonary capillary wedge pressure (PCWP) 10 mmHg, cardiac output 3.4 L/min, and pulmonary vascular resistance (PVR) 14.4 Wood units. The 6-minute walk distance (6MWD) was only 300 meters. Bosentan 250 mg was initiated with oxygen supplementation in January 2006, with subtle improvement of exertional dyspnea. After summer of 2007, her symptom gradually worsened again. In addition, she experienced low-grade fever, loss of appetite, and body weight loss (−5 kg/6 months) with cervical lymphoadenopathy and hepatosplenomegaly, which had worsened despite the use of low-dose prednisolone. She was referred to our hospital for additional evaluation into the etiology of PAH in April 2008.

**Figure 1:**
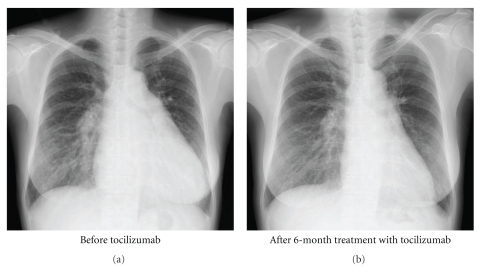
Chest X-ray before tocilizumab and after 6-month treatment with tocilizumab.

She had marked limitation of physical activity (NYHA functional class III), and 6MWD was only 310 meters. Physical examination demonstrated jugular venous dilatation, lower extremity edema, and lymphoadenopathy on cervical, axillary, and inguinal lesions. Nailfold capillary changes were found, but sclerodactyly, muscle weakness, arthritis, and butterfly rash were absent. Laboratory data showed marked anemia (hemoglobin 8.1 g/dL), hypoalbuminemia (2.9 g/dL), polyclonal hypergammaglobulinemia with IgG 6451 mg/dL, CRP 9.1 mg/dL, brain natriuretic peptide (BNP) 181 pg/mL, a positive antinuclear antibody at a titer of 1 : 1,280 with pure speckled pattern, and a positive anti-U1 RNP antibody (86 Index; normal range <15). The protrusion of the main pulmonary artery, increased width of the descending branch of the right pulmonary artery, and an increase in the cardiothoracic ratio were noted on chest X-ray (Figure 1). Electrocardiogram showed signs of an increased right heart load. Hemodynamics assessed by right heart catheterization included man PAP 43 mmHg, PCWP 11 mmHg, right atrial pressure (RAP) 12 mmHg, cardiac output 5.5 L/min, and PVR 5.8 Wood units, indicating that 2-year treatment with bosentan partially improved these parameters. High-resolution computed tomography showed ground-glass opacities with minimal honeycomb cysts on bilateral lower lung field, dilatation of the right atrium, right ventricle, and central pulmonary arteries, and multiple mediastinal lymphadenopathy. Histological evaluation of the biopsied axillary lymph node demonstrated an intense plasmacytosis in the interfollicular areas with a prominent increase in capillaries and postcapillary venules, some of which were hyalinized. These findings were compatible with MCD in mixed histological features of plasma cell and hyaline vascular types [11]. Serology for human immunodeficiency virus (HIV) or human herpesvirus type 8 (HHV-8) was negative. Cytomegalovirus antigenemia was undetectable. Gene sequence for HHV-8 or Epstein-Barr virus was not found in the lymph node. A markedly elevated level of serum IL-6 (41.8 pg/mL; normal range <4) was consistent with the diagnosis of MCD [12]. Thus, we decided to first treat concomitant MCD with tocilizumab at a dose of 8 mg/kg every 2 weeks.

Serial functional, hemodynamic, and laboratory parameters before and after the tocilizumab treatment are summarized in Table 1. After 4 infusions of tocilizumab, low-grade fever and loss of appetite were completely gone, and fatigue was prominently improved with normalization of hemoglobin level. A prominent increase in circulating IL-6 concentration after introduction of tocilizumab indicated efficient IL-6 receptor blockade. Lymphadenopathy and hepatosplenomegaly were gradually resolved and were finally undetectable at 3 months after introduction of tocilizumab. Functional activity was gradually improved to NYHA functional class II and 434 meters at 6MWD. BNP was decreased to 48 pg/mL. Right heart catheterization at 3 months revealed that mean PAP was reduced to 31 mmHg and RAP was 5 mmHg. There was no improvement in cardiac output or PVR, but cardiac output was calculated with the Fick method by an equation containing a hemoglobin level, which rose up markedly after the tocilizumab treatment. In fact, systemic venous oxygen saturation had dramatically improved from 52.1% to 69.4%. Her dyspnea has continued to improve and was finally undetectable (NYHA functional class I) at 6 months when oxygen supplementation was discontinued. The increased right heart load findings on chest X-ray were remarkably improved (Figure 1). After 12 months of treatment with tocilizumab, she was in NYHA functional class I and was able to walk 663 meters at 6MWD without reduction of arterial oxygen saturation. The mean PAP was further decreased to 27 mmHg. The patient has been in NYHA functional class I and in remission of MCD on biweekly tocilizumab, as of May 2010. No side effect of the tocilizumab treatment was apparent.

## 3. IL-6 Overproduction and PAH

MCD is a rare lymphoproliferative disorder characterized by systemic lymphadenopathy and constitutional inflammatory symptoms [11]. Patients with MCD frequently have systemic manifestations, such as low-grade fever, fatigue, loss of appetite, and weight loss. Abnormal laboratory findings include anemia, hypoalbuminemia, hypergammaglobulinemia, and increased acute-phase proteins such as CRP. The etiology of the disease appears to be heterogeneous, but dysregulated overproduction of IL-6 is believed to be responsible for the clinical abnormalities [12]. In fact, IL-6 transgenic mice represented the disease phenotype resembling MCD, which was successfully treated with an anti-IL-6 receptor antibody [13]. In a multicenter prospective study to evaluate the efficacy of tocilizumab in patients with MCD, objective improvement was consistently observed in clinical symptoms, lymphadenopathy and other physical findings, and laboratory parameters [14]. In addition, HHV-8 is reported to be an etiologic agent of MCD, especially in patients infected with HIV [15], since HHV-8 encodes a human IL-6 homolog, which shares functional properties with human IL-6 [16]. 

PAH is a rare complication of MCD [11], and only 4 cases diagnosed with both of these conditions have been reported previously [17–19]. These case reports raise several hypotheses linking PAH and MCD. One hypothesis includes an association of PAH with HHV-8 infection rather than MCD itself. HHV-8 encodes genes homologous to human genes involved in cell proliferation, antiapoptosis, and angiogenesis [20, 21], and HHV-8 gene sequences have been found in plexiform lesions derived from some patients with idiopathic PAH [22], suggesting the possibility that HHV-8 could be involved in the misguided angiogenesis characteristic of PAH. However, HHV-8 infection was not detected in our case as well as in another 2 reported cases complicating MCD and PAH [17, 19]. One of the reported case infected with HIV and HHV-8 showed an unusual complete reversibility of both MCD and severe PAH, with an immunosuppressive treatment with cyclophosphamide, together with highly active antiretroviral therapy and epoprostenol [18]. In addition, tocilizumab induced partial remission of both MCD and PAH in our case as well as in the other HHV-8-uninfected case [19]. These case reports raise another hypothesis that IL-6 is a common pathogenic factor in both MCD and PAH. 

Patients with idiopathic PAH are consistently found to have an increased level of IL-6 in circulation [23, 24] and in lung tissue [25]. In patients with lupus, MCTD, or SSc, a higher serum IL-6 level was reported in patients with PAH than in those without PAH [26–28]. In addition, elevated serum IL-6 was reported in patients with POEMS (polyneuropathy, organomegaly, endocrinopathy, monoclonal gammopathy, and skin changes) syndrome, a rare variant of plasma cell dyscrasia, which sometimes complicates PAH [29]. Several animal models of PAH, including chronic hypoxia [30] and monocrotaline treatment [31], are also associated with increased production of IL-6. Moreover, daily subcutaneous injection of recombinant IL-6 in rats induced the medial thickness of small pulmonary arteries, leading to PAH [32]. These findings together suggest that PAH development is associated with IL-6 overproduction.

## 4. Roles of IL-6 in Pathogenesis of PAH

IL-6 is a pleiotropic cytokine with a wide range of biologic activities in immune regulation, hematopoiesis, inflammation, and oncogenesis [33]. Accumulating evidence indicates pathological roles for IL-6 in various disease conditions, such as inflammatory, autoimmune, and neoplastic diseases. Pathologic features in patients with PAH are characterized by muscularization of distal pulmonary arterioles, concentric intimal thickening, and obstruction of the vascular lumen by proliferating endothelial cells to form plexiform lesions [34]. It has been proposed that dysregulated cellular growth and apoptosis are responsible for a typical proliferative cellular phenotype, resulting in pulmonary vascular remodeling in PAH. On the other hand, perivascular infiltration of inflammatory cells, consisting of T cells, B cells, and macrophages, are often present within and around the affected pulmonary arteries of patients with PAH, suggesting that cytokines and growth factors secreted from these inflammatory cells may be involved in uncontrolled proliferation of pulmonary artery smooth muscle and endothelial cells [35].

**Figure 2:**
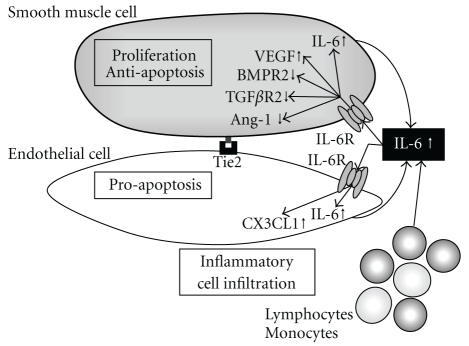
Hypothetical mechanism leading to pulmonary vascular remodeling via overexpression of IL-6. IL-6 induced proliferation and antiapoptosis in vascular smooth muscle cells through upregulation of VEGF, and downregulation of BMPR2 and TGF*β*R2. Upon IL-6 exposure, endothelial cells undergo apoptosis through repressed Tie2 signaling via downregulated Ang-1 expression in smooth muscle cells. Production of CX3CL1 results in recruitment of inflammatory cells, such as lymphocytes and monocytes, which produce enormous amount of IL-6, while vascular smooth muscle and endothelial cells also produce IL-6 upon stimulation with IL-6.

In this regard, a lung-specific overexpression of IL-6 in mice resulted in increased PVR and pathological lesions similar to that seen in patients with PAH, including distal arteriolar muscularization, plexogenic arteriopathy, and periarteriolar infiltration of T cells [36]. These findings indicate that IL-6 directly or indirectly promotes proliferation of both smooth muscle and endothelial cells, which are potentially mediated through a number of proliferative, prosurvival, and anti-apoptotic processes (Figure 2). In this regard, IL-6 triggers vascular smooth muscle cell proliferation through upregulated expression of vascular endothelial growth factor (VEGF) and its receptor VEGFR2 [36, 37], which was observed in the plexiform lesions of patients with PAH [38]. 

It has been known that transforming growth factor (TGF)-*β*/bone morphogenetic protein (BMP) signaling controls growth of vascular smooth muscle and endothelial cells by inhibiting excessive proliferation. Genetic mutations in the gene encoding the type II receptor of BMP (BMPR2) comprise a genetic hallmark of heritable PAH [39], and downregulated protein expression of BMPR2 has also been described in nonheritable PAH [40]. A recent study by Brock et al. demonstrated that IL-6 repressed protein expression of BMPR2 through overexpression of microRNA cluster 17/92 [41]. MicroRNAs regulate posttranslational mechanisms by binding to their target mRNAs and by altering mRNA stability or affect protein translation [42]. Interestingly, protein expression of TGF*β*R2, another receptor from the identical protein family, was also modulated by the same microRNA cluster [43]. This may explain the lack of TGF*β*R2 expression in plexiform lesions of patients with PAH [44]. The promoter region of the microRNA 17/92 gene C13 or f25 has a highly conserved binding site for STAT3, a major IL-6 signal transduction pathway [33]. In fact, persistent activation of STAT3 resulted in repressed protein expression of BMPR2 [41]. 

Angiopoietin-1 (Ang-1)-Tie2 pathway is essential for both embryonic and postnatal angiogenesis and involves in a protective action on endothelial cells by suppressing inflammation and apoptosis [45]. The role of this system in the pathogenesis of PAH has been poorly understood, but a recent study using Tie2-deficient mice found that endothelial survival signaling via the Ang-1-Tie2 pathway is protective in PAH [46]. Exposure of IL-6 decreased expression of Ang-1 in lung vascular smooth muscle cells, leading to reduction of Tie2 activity in endothelial cells and resultant excessive apoptosis. 

On the other hand, a constitutive activation of STAT3 was described in vascular smooth muscle cells as well as in endothelial cells from the lung tissue of patients with PAH [47]. Upon stimulation with IL-6, endothelial cells produce CX3CL1/fractalkine, a potent chemokine that recruits monocytes and lymphocytes into the lung [48]. These mononuclear infiltrates are major source of IL-6, but vascular smooth muscle cells and endothelial cells also produce IL-6 upon stimulation with IL-6 [36, 49]. Taken together, IL-6 promotes the development and progression of pulmonary vascular remodeling, leading to PAH, via a variety of mechanisms.

## 5. The “Second-Hit” Process for Developing PAH

However, PAH observed in mice undergoing IL-6 overexpression was subtle, but was hemodynamically and histologically remarkable upon hypoxia exposure [36, 50]. In contrast, IL-6 knockout mice exposed to hypoxia were resistant to the development of increased PVR [51]. This is consistent with the “second-hit” theory for the pathogenesis of PAH, in which the response to an environmental or endogenous trigger is enhanced in susceptible individuals [52]. For example, exposure to serotonergic or inflammatory stressors produced an enhanced pulmonary hypertensive response in BMPR2 deficient mice [53]. Therefore, it is likely that overproduction of IL-6 alone has minimal impact on development of PAH, but the combination with other factors known to enhance susceptibility to pulmonary vascular remodeling, such as hypoxia and vasculopathy in CTD, appears to have synergistic effects resulting in the development of significant PAH.

The contribution of IL-6 to the pathogenesis of PAH may be different among associated conditions. In this regard, circulating IL-6 level was increased in patients with SSc, and the increased level was associated with the presence of PAH [28, 54]. In addition, the IL-6 level in bronchoalveolar lavage fluid was also increased in patients with SSc irrespective of the presence or absence of interstitial lung disease [55]. IL-6 is abundantly produced *in vitro* by affected skin fibroblasts and alveolar macrophages derived from patients with SSc [56, 57]. These findings together indicate that the lung tissue of SSc patients is always exposed to IL-6. This may explain why prevalence of PAH is the most frequent in patients with SSc among CTDs.

## 6. Summary and Future Perspectives

Current therapeutic strategies for PAH focus on the pulmonary vascular endothelium and its role in regulating smooth muscle cell tone and proliferation. By using these modern PAH drugs, treatment of PAH has undergone an extraordinary evolution even in patients with CTD, but PAH still remains a chronic intractable disease without a cure. A better understanding of the underlying pathophysiology of the pulmonary vasculature is needed for better therapy. In this regard, the findings of pathologically aberrant proliferation of smooth muscle and endothelial cells as well as increased expression of secreted growth factors, such as VEGF and platelet-derived growth factor (PDGF), in PAH have caused a shift in paradigm in treatment strategies for this disease [58]. The efficacy of imatinib, a prototypical PDGF receptor signaling inhibitor, was reported in patients with severe PAH [59–61], and a phase II multicenter clinical trial of imatinib in patients with PAH has been completed in the United States and Europe and the results are pending. On the other hand, our case report indicates that IL-6 is another potential therapeutic target for PAH. 

In summary, this report describes our experience with the use of tocilizumab in a patient with MCTD and PAH. The rationale for such treatment derives from a numerous basic studies showing a critical role of IL-6 in the promotion of pulmonary vascular remodeling and consequent development of PAH. The concept underlying use of IL-6 blockade in PAH is prevention and reversal of lung vascular remodeling rather than prolonged vasodilation of pulmonary arteries. We recognize the limitations of a single case report, but we believe that blockade of IL-6 signaling may be a promising new therapy for PAH, especially in the context of CTD. Further studies of IL-6 blockade in PAH patients with and without CTD are warranted.

## Figures and Tables

**Table 1:** Serial functional, hemodynamic, and laboratory parameters before and after the tocilizumab treatment.

	Pretreatment	3 months	6 months	9 months	12 months
NYHA functional class	III	II	I	I	I
6MWD (m)	310	434	ND	ND	663
Mean PAP (mmHg)	43	31	ND	ND	27
PCWP (mmHg)	11	4	ND	ND	4
RAP (mmHg)	12	2	ND	ND	3
Systemic venous oxygen saturation (%)	52.1	69.4	ND	ND	75.3
Cardiac output (L/min)	5.5	4.5	ND	ND	4.4
PVR (wood unit)	5.8	5.6	ND	ND	5.3
Doppler systolic PAP (mmHg)	100	90	72	51	54
BNP (pg/mL)	181	48	44	46	37
CRP (mg/dL)	9.01	0.54	0.25	0.12	0.04
IgG (mg/dL)	6,451	3,266	2,679	2,433	2,238
Hemoglobin (g/dL)	8.0	12.9	13.9	13.0	12.8
IL-6 (pg/mL)	41.8	1,100	801	806	756

ND, 6MWD and hemodynamic assessment by right heart catheterization were not done at 6 and 9 months.
